# Changes in symmetry of anterior chamber following routine cataract surgery in non-glaucomatous eyes

**DOI:** 10.1186/s40662-019-0144-3

**Published:** 2019-07-03

**Authors:** Hanbin Lee, Ieva Zukaite, Valerie Juniat, Maria E. Dimitry, Amanda Lewis, Mayank A. Nanavaty

**Affiliations:** Sussex Eye Hospital, Brighton & Sussex University Hospitals NHS Trust, Eastern Road, Brighton, BN2 5BF UK

**Keywords:** Anterior chamber depth, Anterior chamber angle, Angle to angle diameter

## Abstract

**Background:**

To assess minimum and maximum changes in anterior chamber dimensions following routine cataract surgery in non-glaucomatous eyes.

**Methods:**

Forty-two eyes (42 participants) underwent routine cataract surgery with same surgeon and were assessed preoperatively, 1, 3, 6 and 12 months postoperatively. Primary outcome measure: Angle-to-angle diameter (AAD) (at 0-180^o^, 45-225^o^, 90-270^o^, 135-315^o^), Anterior-chamber-angle (ACA) (at 0^o^, 45^o^, 90^o^, 135^o^, 180^o^, 225^o^, 270^o^ and 315^o^) and central anterior chamber depth (ACD) at all visits. Secondary outcome measures: relationship to axial length (AL).

**Results:**

The mean AAD and ACA increased post-operatively in all meridians at all visits postoperatively. At 12 months, there was a maximum change in AAD in horizontal meridian (506.55 ± 468.71 μm) and least in vertical meridian (256.31 ± 1082.3 μm). The mean percentage increase in ACA postoperatively was least at 90^o^ (5% increase compared to 29–35% elsewhere). Central ACD deepened at all postoperative visits and this did not change over 12 months. There was no correlation between AAD, ACA and ACD with AL at any visit.

**Conclusion:**

The AAD, ACA and ACD increases following cataract surgery in non-glaucomatous eyes, but at 12 months increase in AAD is least in vertical compared to horizontal meridian. Also, ACA was narrower (only 5% increase) superiorly compared to elsewhere (29–35% increase in ACA). This may have implications with regards to surgeries performed in the anterior chamber and corneal endothelial cell loss.

## Background

Cataract surgery can be combined with procedures needing foreign objects such as glaucoma tubes and secondary intraocular lenses (IOLs) in the anterior chamber. It is also established that glaucoma tubes [1–6] and secondary intraocular lenses (IOLs) [7] can lead to long-term endothelial cell loss. Secondary anterior chambers IOLs are placed in a particular axis and they are large enough to cover a significant area in the anterior chamber. However, glaucoma drainage devices e.g., Baerveldt, Ahmed and Molteno tubes, which are most commonly placed in supero-temporal anterior chamber angle and despite covering a relatively smaller area in the anterior chamber (and not covering a significant area of the anterior chamber, unlike a secondary IOL) are reported to reduce the endothelial cell count in the long term. Moreover, it is established that the distance of the tube to the endothelium may be a contributing factor to the reduction of endothelial cell count (ECC). We know that routine cataract surgery is associated with a post-operative increase in anterior chamber angle and anterior chamber depth [8]. However, most of these studies have reported average change in anterior chamber parameters, but there is a paucity of literature on further details as to whether these changes are uniform across the anterior chamber or they are maximum/minimum in certain areas of anterior chamber following cataract surgery.

There are several methods available for measuring changes in anterior chamber parameters [9, 10]. The Oculus Pentacam HR® (Pentacam, Oculus, Wetzlar, Germany) is a combined anterior segment imaging device consisting of a slit illumination system and a single rotating Scheimpflug camera which revolves around the eye [11]. The device offers a rapid, non-invasive method of evaluating the anterior segment from the anterior corneal surface to the posterior lens surface [11–13]. In particular, it can be used to calculate keratometry and anterior chamber measurements [13]. Changes in the anterior segment can be subsequently followed with repeated exams [12]. Studies have demonstrated good repeatability of measurements with Pentacam for anterior segment measurements [14].

The aim of this study was to identify parts of the anterior chamber where maximum and minimum changes occur with angle-to-angle diameter in horizontal, vertical and oblique meridians, anterior chamber angle at various degrees and with central anterior chamber depth following standard cataract surgery in non-glaucomatous eyes with posterior chamber intraocular lens implantation using a Scheimpflug analyser.

## Methods

This was an observational study performed on patients who were part of a prospective, randomised controlled study at the Sussex Eye Hospital, Brighton, United Kingdom (Limbal relaxing incision [LRI] vs Toric IOL for corneal astigmatism during cataract surgery - UKCRN ID: 16848; ClinicalTrials.gov: NCT02067429) between June 2013 and March 2015 [15]. The study protocol was reviewed and approved by the ethics committee (ref 14/LO/0440). The study followed the tenets of the Declaration of Helsinki.

Inclusion criteria were symptomatic cataract for which the patient desires surgery and corneal astigmatism of ≥0.75 D and ≤ 2.5 D on topography. Exclusion criteria were < 18 years of age, significant ophthalmic comorbidity detrimental to final visual outcomes, unable to give consent for surgery and research, concurrent use of ocular medications including lubricants, unable to attend follow up visits for research purposes and any complications at the time of surgery needing any additional intraoperative procedure/s. Written informed consent was obtained from each patient and the nature of the study was explained before assessments.

Patients underwent standard, uncomplicated cataract surgery with phacoemulsification and IOL implantation. The same surgeon carried out the procedures via a superior corneal approach with an incision width of 2.75 mm. All patients received the same IOL design (C-flex or T-flex IOLs, Rayner, Worthing, UK). Astigmatism was either corrected using a toric T-flex IOL or a peripheral corneal relaxing incision (PCRI). PCRI was calculated using www.lricalculator.com prior to the surgery [15]. The details of the surgical methodology are published elsewhere [15].

Patients were assessed preoperatively and at 1, 3, 6 and 12-month follow-up visits after surgery. If the participant missed only a single follow up appointment out of 4 postoperatively (1, 3, 6 and 12 months) the data was still collected for analysis. A single eye (first eye) of all patients was included as per this study protocol. At all visits, Scheimpflug imaging using the Pentacam HR® (Oculus, Germany) was performed on patients by a single experienced ophthalmic technician. The device uses a high-resolution, 1.45 M pixel camera that captures 138,000 data points in fewer than 2 s. A 475 nm wavelength blue light-emitting diode and the camera rotate together around the optical axis to obtain anterior segment images [13]. Three scans were taken for each eye and the scan with no artefact or no quality issues and where the entire front of the eye was visible was saved on the computer attached to the Scheimpflug device for the analysis. Scans with eyelid artefacts were not saved. The patients were asked not to blink or to widen their palpebral aperture unnaturally while performing the scans.

**Fig. 1:**
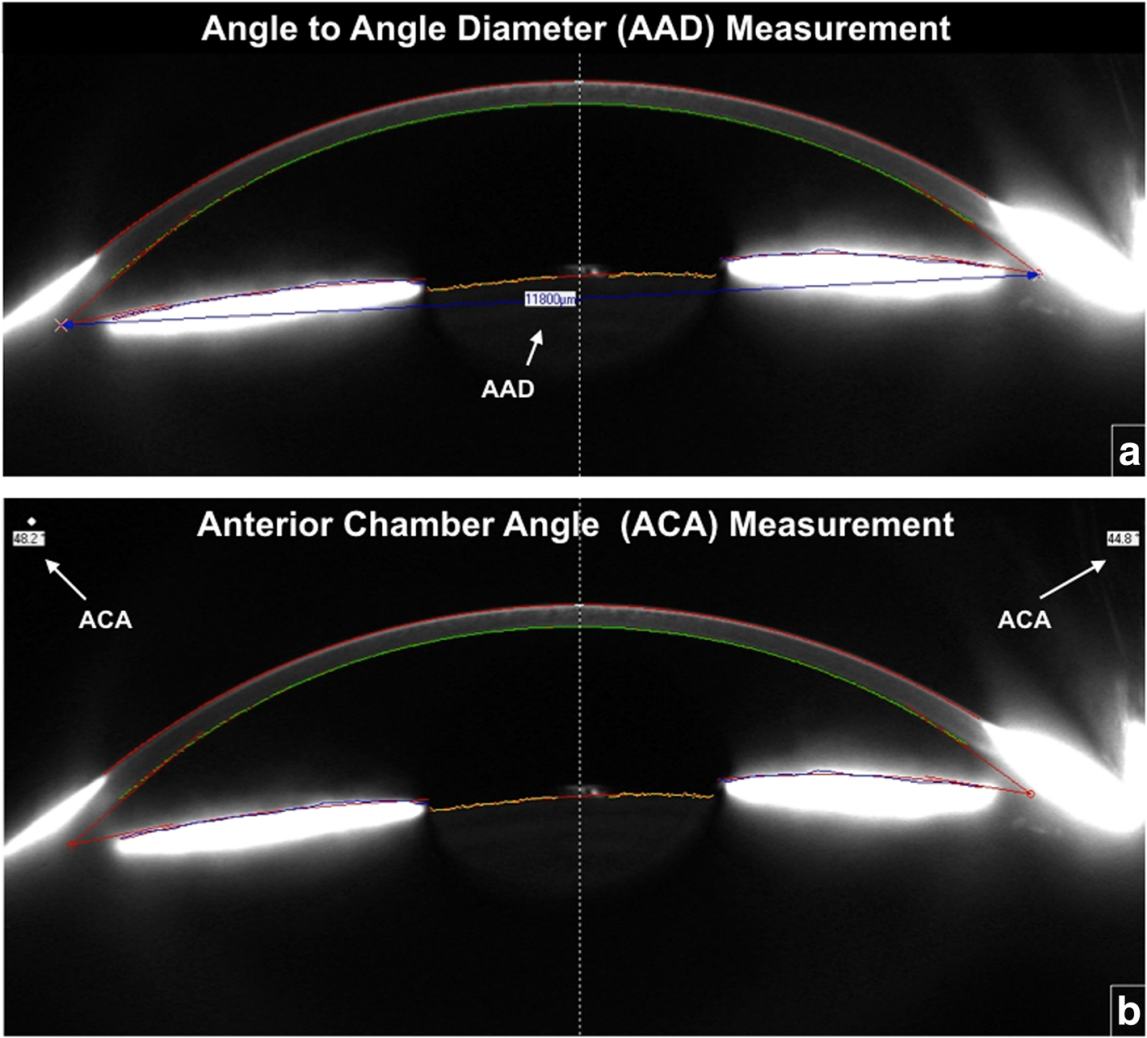
Measurements of angle-to-angle diameter (AAD) and anterior chamber angle (ACA). **a**. The measurement of angle-to-angle diameter on Pentacam Software. **b**. The measurement of anterior chamber angle on Pentacam Software

Demographic data was collected from paper records and included age, date of surgery, and date of pre-operative and post-operative visits. Axial length measurements were obtained using optical biometry (IOLMaster®, Carl Zeiss, Germany). Anterior chamber parameters recorded on the Pentacam HR® included:

1. *Angle-to-angle diameter:* This was measured manually using the software’s callipers and was defined as a line joining the scleral spurs on the Scheimpflug images (Fig. 1a). For this measurement, the point of intersection of the iris and posterior corneal surface was defined as scleral spur. Angle-to-angle diameter (AAD) was measured at 4-184^o^ (horizontal), 42-222^o^ (oblique), 91-271^o^ (Vertical), 137-317^o^ (oblique) (Fig. 1a) (to simplify the analysis, above axes were represented as 0-180^o^, 45-225^o^, 90-270^o^, 135-315^o^).
2. *Anterior chamber angle:* The Pentacam HR® software automatically generated an anterior-chamber-angle (ACA) size at each angle (Fig. 1b). The Scheimpflug camera on the Pentacam HR® machine captures several Scheimpflug images at different degrees of rotation. The ACA measurements at different meridians were noted on the images and this was further verified using a protractor on the image displayed on the Pentacam. Care was taken to correctly identify the scleral spur and the anterior surface of the iris in each image. This was measured at 0^o^ (horizontal), 45^o^ (oblique), 90^o^ (vertical), 135^o^ (oblique), 180^o^ (horizontal), 225^o^ (oblique), 270^o^ (vertical) and 315^o^ (oblique).
3. *Anterior chamber depth (ACD):* The central ACD was measured manually using the Pentacam HR® software’s digital measuring tool and was defined as the axial distance from the posterior corneal surface to the lens surface measured at the pupil centre.

### Statistical analysis

All data was recorded on Microsoft Office Excel® 2016 (Microsoft® Corporation, USA). Normality of all the data was tested by Kolmogorov-Smirnov test. The SPSS statistics version 22.0 (International Business Machines® Corporation) and Microsoft Office Excel® 2016 (Microsoft® Corporation, USA) was used for all statistical analysis. The ANOVA test was used to compare all the parameters during all visits (pre-operatively and post-operatively at 1, 3, 6 and 12 months). The t-test was then used to compare all parameters between the preoperative assessment and at each visit. A *P* value of < 0.05 was considered statistically significant.

To reduce the bias in the subjective measurements, two authors (HL and IZ) collected the data on all measurements. The study by Patton et al. [16] was used as a guide for selection of the statistical methods for assessing agreement between the two observers. To predict the overall agreement between the two authors, repeatability was only assessed using intraclass correlation coefficient and coefficient of repeatability for AAD measurement at 0-180^o^ meridian only.

## Results

Forty-two patients (42 eyes) were included. None of the patients were excluded due to any intraoperative complications needing additional intraoperative procedure/s. The average age was 73.97 ± 10.71 years (range: 51–90 years). Average axial length was 23.75 ± 1.28 mm (range: 21.67–27.03 mm). Observers HL and IZ showed good agreement in AAD measurements in the horizontal meridian at 0-180^o^, which we employed as a sample; intra-class coefficient being 0.834 and the coefficient of repeatability being 725.83 (2 × standard deviation of differences).

### Angle to angle diameter

The mean AAD significantly increased post-operatively in all meridians at 1, 3, 6 and 12 months (Table 1). At 12 months, we found the maximum increase in AAD in the horizontal meridian and least increase in the vertical meridian (Table 1). There was no statistically significant change in AAD between 1 month and 12 months in all four meridians (Table 1). One-way ANOVA showed statistically significantly different AAD means pre-operatively, 1, 3, 6 and 12 months (Table 1). There was no correlation between axial length and AAD change at all meridians upon regression analysis (Table 2).

**Table 1:** Angle-to-angle diameter (AAD), anterior chamber angle (ACA) and central anterior chamber depth (ACD) pre and postoperatively at all visit

	Pre-operative (mean ± SD) (range)	1 month (mean ± SD) (range)	Preoperative vs. 1 month (*P* value)^^^	3 months (mean ± SD) (range)	Preoperative vs. 3 month (*P* value)^^^	6 months (mean ± SD) (range)	Preoperative vs. 6 month (*P* value)^^^	12 months (mean ± SD) (range)	Preoperative vs. 12 month (*P* value)^^^	Mean change in AAD at 12 months (mean ± SD) (range)	Preoperative vs. all follow up visits (*P* value)^$^
Angle-to-angle diameter (AAD) (microns)											
0-180^o^ meridian	11210.47 ± 560.65 (9820–12,380)	11510.71 ± 606.23 (10315–13,285)	0.00*	11577.98 ± 582.19 (10645–13,225)	0.00*	11515.95 ± 521.0 (10610–12,940)	0.00*	11551.42 ± 494.23 (10570–12,485)	0.00*	506.55 ± 468.71 (140–1070)	0.02*
45-225^o^ meridian	11064.28 ± 606.04 (9630–12,520)	11358.93 ± 582.49 (9580–12,480)	0.00*	11430 ± 100.78 (6590–13,640)	0.00*	11422.98 ± 489.32 (10570–12,195)	0.00*	11466.80 ± 471.01 (10695–12,575)	0.00*	402.5 ± 448.28 (360–1325)	0.04*
90 – 270^o^ meridian	10913.21 ± 720.85 (8905–12,410)	11325.24 ± 632.72 (9770–13,035)	0.00*	11443.45 ± 582.99 (10430–13,460)	0.00*	11407.14 ± 546.49 (10315–12,340)	0.00*	11169.52 ± 1147.51 (6565–12,775)	0.00*	256.31 ± 1082.3 (4430–1960)	0.01*
135-315^o^ meridian	11067.26 ± 594.80 (9575–12,240)	11534.88 ± 641.82 (10275–12,925)	0.00*	11526.90 ± 565.59 (10325–12,895)	0.00*	11487.02 ± 586.83 (10270–12,845)	0.00*	11573.81 ± 586.04 (10290–12,735)	0.00*	340.95 ± 314.94 (640–1240)	0.00*
Anterior chamber angle (ACA) (degrees)											
0^o^	39.41 ± 16.90 (18.1–86.1)	46.14 ± 6.38 (34.6–60.6)	0.01*	46.88 ± 7.02 (30.6–65.2)	0.00*	47.40 ± 7.26 (30.9–64.7)	0.00*	46.56 ± 7.41 (27.0–62.0)	0.01*	7.15 ± 17.11 (− 45.7–25.8)	0.00*
45^o^	38.72 ± 15.20 (13.1–83.1)	42.06 ± 10.07 (27.0–77.7)	0.25	41.85 ± 9.30 (27.5–87.5)	0.29	40.30 ± 6.27 (26.1–58.5)	0.54	41.03 ± 5.94 (28.1–57.6)	0.36	2.31 ± 16.08 (− 46.4–27.1)	0.54
90^o^	40.02 ± 19.26 (17.3–97.6)	40.26 ± 12.74 (24.3–89.8)	0.26	39.04 ± 11.19 (25.1–89.9)	0.18	37.32 ± 8.80 (23.7–63.7)	0.06	38.70 ± 8.38 (24.9–65.7)	0.12	−5.32 ± 21.27 (− 60.5–21.1)	0.15
135^o^	37.78 ± 16.81 (13.2–73.9)	38.99 ± 6.27 (26.4–49.4)	0.63	40.87 ± 6.95 (26.0–62.0)	0.26	40.01 ± 5.95 (29.5–59.5)	0.40	41.02 ± 6.69 (25.9–58.8)	0.23	3.23 ± 17.12 (− 28.7–28.6)	0.49
180^o^	38.76 ± 12.92 (20.0–75.1)	46.95 ± 6.44 (32.4–60.7)	0.00*	46.74 ± 6.74 (32.1–62.7)	0.00*	47.63 ± 5.99 (36.7–59.6)	0.00*	47.46 ± 6.34 (33.9–62.8)	0.00*	8.70 ± 11.56 (− 24.1–27.6)	0.00*
225^o^	39.53 ± 15.02 (20.3–72.4)	45.56 ± 6.63 (34.5–63.6)	0.02*	46.03 ± 5.55 (33.3–50.3)	0.01*	45.71 ± 7.14 (32.6–64.7)	0.02*	45.77 ± 7.35 (33.9–75.2)	0.02*	6.24 ± 18.51 (− 30.8–48)	0.00*
270^o^	38.90 ± 18.15 (16.5–102)	41.37 ± 5.27 (30.5–53.5)	0.41	41.34 ± 5.25 (30.1–51.8)	0.44	40.35 ± 5.30 (29.3–51.5)	0.64	41.71 ± 5.76 (28.5–54.9)	0.35	2.80 ± 19.43 (− 59.1–33.6)	0.65
315^o^	38.64 ± 13.58 (17.2 – 92.4)	45.70 ± 6.94 (31.9 – 68.5)	0.00*	45.45 ± 7.45 (29.7 – 70.5)	0.00*	45.63 ± 7.35 (27.2 – 64.1)	0.00*	46.32 ± 8.55 (27.8 – 73.9)	0.00*	7.68 ± 15.01 (− 50.9 – 36.2)	0.00*
Anterior chamber depth (ACD) (mm)											
	2.57 ± 0.41 (1.92–3.54)	4.05 ± 0.49 (2.72–5.07)	0.00	4.14 ± 0.45 (3.14–5.51)	0.00	4.01 ± 0.43 (3.06–4.88)	0.00	4.08 ± 0.43 (3.31–5.19)	0.00	1.51 ± 0.48 (0.38–2.89)	0.00

^^^Paired t test, ^*^ Statistically significant, ^$^ANOVA test, *SD* = standard deviation

**Table 2:** Regression analysis: Axial length versus change in AAD, change in ACA and central ACD postoperatively at 12 months

Meridian (Degrees)	R square Value	*P*-Value
Angle-to-angle diameter (AAD)		
0–180	0.022	0.34
45–225	0.094	0.05
90–270	0.037	0.22
135–315	0.034	0.24
Anterior chamber angle (ACA)		
0	0	0.94
45	0.03	0.27
90	0.01	0.46
135	0	0.74
180	0.03	0.29
225	0.02	0.33
270	0	0.7
315	0	0.92
Anterior chamber depth (ACD)		
	0.001	0.00

### Anterior chamber angle

**Fig. 2:**
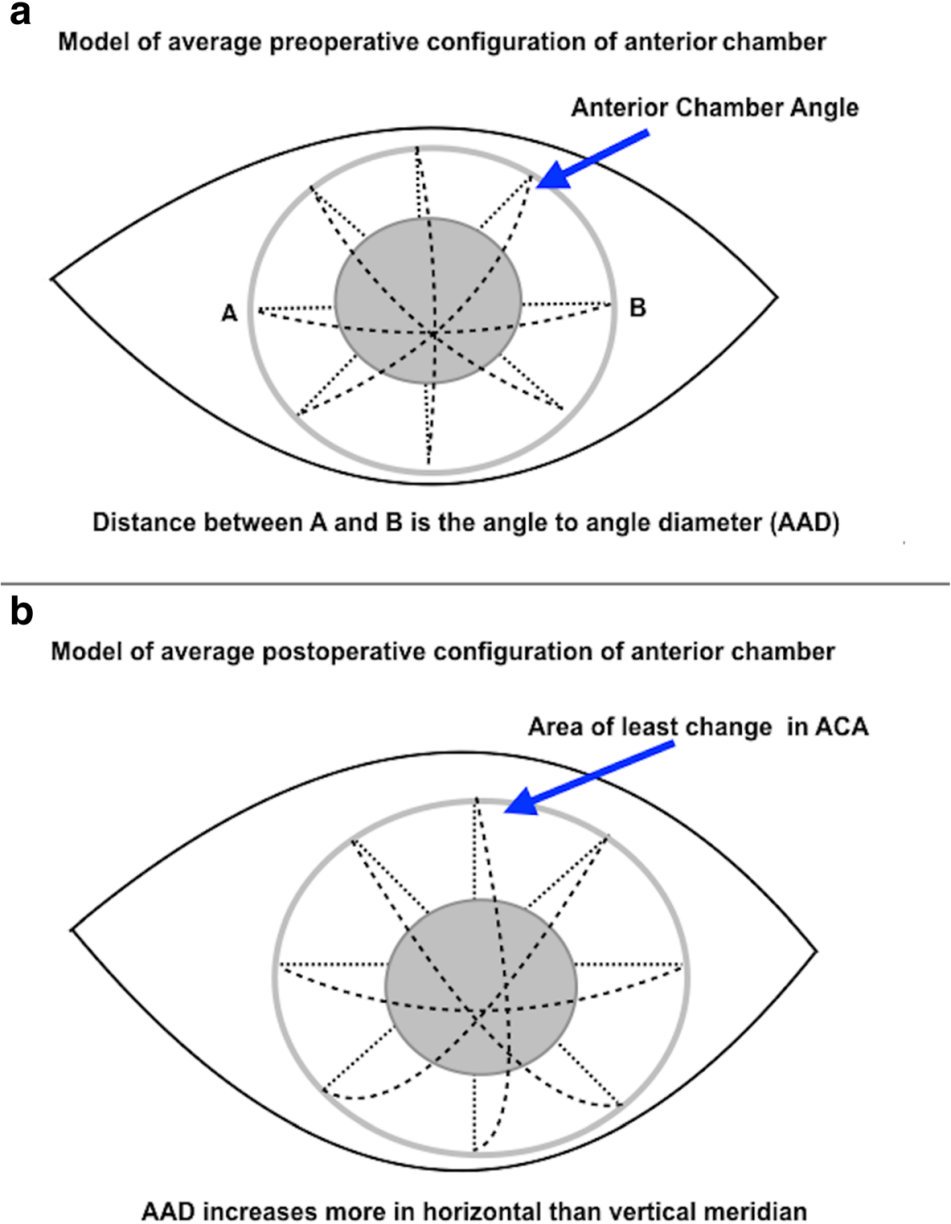
Dimensions of anterior chamber preoperatively and postoperatively. **a**. Diagram of the preoperative structure of the anterior chamber. **b**. Diagram of the postoperative structure of the anterior chamber

The mean ACA increased at all degrees (Table 1). This was significant at 0^o^, 180^o^, 225^o^ and 315^o^ postoperatively at month 1, 3, 6 and 12 (*P* < 0.05, Table 1). The mean percentage increase in ACA at 12 months post operatively was 35.05, 22.53, 5.71, 32.10, 32.62, 34.41, 29.38 and 32.57% at 0^o^, 45^o^, 90^o^, 135^o^, 180^o^, 225^o^, 270^o^ and 315^o^, respectively. One-way ANOVA also showed significant differences in ACA at pre op, 1, 3, 6 and 12 months at horizontal and infero-lateral degrees (0° and 180°) and at two inferior degrees 225° and 315°. Postoperatively, there was least deepening of anterior chamber noted at 90° (Fig. 2a and b). There was no correlation between axial length and ACA change on linear regression analysis (Table 2).

### Central anterior chamber depth (ACD)

The mean central ACD measurement increased significantly post-operatively at months 1, 3, 6 and 12 (Table 1). There was no correlation between changes in ACD post-operatively on linear regression (Table 2).

## Discussion

It is already known that ACA and ACD increase following cataract surgery [17–20]. However, it was not known whether this increase was symmetrical in all quadrants and meridians of the anterior chamber. We found that the AAD, ACA and ACD increases following cataract surgery in non-glaucomatous eyes, but AAD is narrower in vertical compared to horizontal meridian. Also, ACA was narrower (only 5% increase) superiorly compared to elsewhere (29–35% increase in ACA).

There is little evidence in the literature reporting changes in AAD following cataract surgery. We studied these changes in various meridians pre- and post-operatively in the same non-glaucomatous eyes undergoing cataract surgery with a standardized technique by the same surgeon. In our patients, we found the maximum increase in AAD in the horizontal meridian and least increase in the vertical meridian, postoperatively (Table 1), which is in accordance with other studies [21, 22]. Some studies [21–23] have used ultrasound to measure the AAD. Rondeau et al. [21] scanned 28 eyes of 14 subjects with ultrasonography and found a general trend for orientation of the meridian of largest diameter to be in the horizontal meridian. Werner et al. [22] found that mean values of the AAD at the vertical meridian were significantly higher than that at the horizontal meridian with ultrasonography. Petermeier et al. [23] found the vertical AAD was significantly larger than the horizontal diameter when measured using very high frequency ultrasound (VHF-US, Artemis, Canada). Other studies [22, 24] used OCT to measure AAD. Werner et al. [22] found that the vertical meridian was significantly lower than that of the horizontal meridian with anterior segment OCT. Baikoff et al. evaluated the AAD with a different commercially available anterior segment ocular coherence tomography (AS-OCT) system (Carl Zeiss, Meditec) [25]. In their series of 89 phakic eyes, the vertical diameter was at least 100 μm larger than the horizontal diameter in 74% of the cases [25]. The reason why AAD increase less vertically postoperatively in our and some other studies is still unknown. Based on the findings of Werner et al. [22], the anterior chamber is suggested to be oval, so we hypothesize that removal of the centripetal forces from the zonules of the round, bulky cataractous lens, combined with the creation of space in the anterior segment of the eye, compounded with eyelid blinking, may be responsible for relaxing all the centripetal forces, which in turn lead to changes in AAD increasing less in the vertical meridian compared to the horizontal meridian (Fig. 2a and b).

ACA has been shown to increase following cataract surgery on ultrasound biomicroscopy [26–28] and on AS-OCT [24]. In a study by Kurimoto et al. [26] using ultrasound biomicroscopy, they concluded that the narrower the preoperative angle was to begin with, the greater the postoperative change of the angle following cataract surgery. As stated by Kurimoto et al. [26], whereas the iris in phakic eyes was in contact with the lens, the iris in pseudophakic eyes was free from IOL contact, as long as it was implanted in the capsular bag. The pupillary plane shifted backwards from the anterior chamber wall, deepening the anterior chamber by approximately 850 μm [26]. Hayashi et al. [18] using Scheimpflug video photography*,* showed that the mean ACA in angle-closure patients became almost identical to those found in open-angle glaucoma patients and in non-glaucomatous eyes following cataract extraction. Our study shows that there is less increase in ACA superiorly compared to elsewhere in the anterior chamber (Table 1). Our findings are more detailed compared to the above studies as the previous studies report the difference in mean values for the entire anterior chamber whereas we reported differences at each meridian in the anterior chamber.

Our study showed that the ACD significantly increased following cataract extraction. Central ACD has been shown on ultrasound biomicroscopy to increase following cataract surgery [26–28]. In particular, Kurimoto et al. [26] found a greater postoperative change in eyes with shallower anterior chambers. Similarly, Shin et al. [20] also reported a significant increase in mean ACD postoperatively for their patients with occludable angles and found that anterior chamber deepening was inversely related to preoperative anterior chamber depth. These findings have also been reported in studies using AS-OCT [10, 17, 29]. An increase in ACD following cataract extraction is elegantly explained by Kurimoto et al. [26] as explained above.

There are several clinical implications of our findings. Like our study, Werner et al. [22] confirmed that the anterior segment of the human eye is not geometrically round. Moreover, we also found that the superior anterior chamber is shallower than inferior anterior chamber pre- and post-operatively. This has a direct impact on the choice of the size of angle-fixated IOLs to be implanted as well as of the best site (meridian) for the fixation of these IOLs in each eye. Baikoff [24] recommended adapting angle-supported IOLs to the largest internal diameter of the anterior chamber and inserting them along this axis to avoid the propeller effect (spinning of the IOL in the anterior chamber on Z axis), which occurs when the IOL is smaller than the axis on which it has been placed. He also considers the choice of the size of the posterior chamber IOL to be implanted in the ciliary sulcus to be a more complex issue. We found AAD to be largest horizontally, but anterior chamber is shallower superiorly also. Thus, placement of the anterior chamber IOL in the longest diameter of the anterior chamber (i.e. horizontal diameter) after appropriate sizing should lead to more stability of these IOLs.

It should be noted that the anterior chamber angle is narrower superiorly, which will increase the proximity of the secondary IOL to the corneal endothelium superiorly, if placed vertically in the anterior chamber. In such a situation, if the sizing is inadequate, the likelihood of extensive endothelial cell loss increases, leading to corneal decompensation. In a study by Alio et al. [7], corneal decompensation was reported to be 24% due to inadequate anterior chamber anatomy. Furthermore, glaucoma surgeons prefer to place the tubes superotemporally when placing the drainage plate. The second most common site is supero-nasal. Occasionally, tubes can be placed infero-temporally or infero-nasally. Inferior siting of the tube and plate is more likely to resolve in exposure and infection [30]. Patients requiring glaucoma drainage devices may be phakic, pseudophakic or aphakic. We found that the superior part of the anterior chamber is narrower in pseudophakic eyes compared with the remainder of the anterior chamber. A 3-year study of Baerveldt tubes by Tan and colleagues [6] on 53 patients found that endothelial cell loss was greatest when the tube-cornea distance decreased and, in the quadrant containing the tube. Endothelial cell density (ECD) loss occurred at a yearly rate of 4.54% centrally and 6.57% in the peripheral quadrant, on average. In cases with shorter tube-cornea distances as measured by anterior segment optical coherence tomography, the observed ECD loss was 6.20% centrally and 7.25% in the peripheral quadrant, compared with 4.11% centrally and 5.77% in the peripheral quadrant ECD loss in eyes with longer tube-cornea distances [6]. Another study of Ahmed valve implants found similar results with greater ECD preservation in eyes with a greater tube-cornea distance [1]. In contrast, Mendrinos and colleagues [4] used anterior segment optical coherence tomography on 10 patients to measure tube-cornea distance but did not find any association of endothelial cell loss with tube-cornea, tube-iris, or intracameral length of the drainage tube. That study also compared endothelial cell loss centrally and peripherally but did not find a difference in cell loss (7.9% ± 2.5 and 7.5% ± 2.4%, respectively) [4]. Recently, a relatively new glaucoma stent (Cypass, Alcon, Fort Worth, Texas) was withdrawn due to safety concerns over ECC (https://www.novartis.com/news/media-releases/alcon-announces-voluntary-global-market-withdrawal-cypass-micro-stent-surgical-glaucoma). This minimally invasive glaucoma drainage device is made of a very rigid material and when sited in a more anterior position, may have been in contact with corneal endothelium leading to increased ECC.

The limitation of this study is that the measurements of AAD, ACA and ACD had a subjective element to it. However, to reduce the subjectivity we included two observers (HL and IZ) and calculated the repeatability based on an established method published by Patton et al. [16] A much better approach would be to use the within subject standard deviation (Sw) as a descriptor of reproducibility via the ANOVA method and to determine the reproducibility limits as described by McAlinden et al. [31] In our study only one measurement was taken from one (best) scan out of 3 scans per patient per observer (HL and IZ) and therefore, we were unable to calculate Sw.

## Conclusions

In summary, this study supports the findings that AAD, ACA and ACD change significantly following cataract surgery and anterior chamber is not perfectly symmetrical in geometry in pseudophakic eyes. We further conclude that AAD changes more in horizontal and least in vertical meridians, while ACA increases more inferiorly compared to superiorly. This may help inform positioning of future glaucoma drainage devices or minimally invasive glaucoma stents for maximum efficacy and minimal endothelial cell loss.

## Data Availability

Not applicable.
